# ADAMTS13 Gene Polymorphisms and Coronary Artery Disease Risk, Long-Term Survival, and Risk Factor Profile

**DOI:** 10.3390/genes17050508

**Published:** 2026-04-25

**Authors:** Justyna Wrona, Anna Balcerzyk-Matić, Katarzyna Mizia-Stec, Artur Filipecki, Jolanta Krauze, Paweł Niemiec

**Affiliations:** 1 Department of Biochemistry and Medical Genetics, Faculty of Health Sciences in Katowice, Medical University of Silesia, Medykow Street 18, 40-752 Katowice, Poland; justyna.wrona@sum.edu.pl (J.W.); pniemiec@sum.edu.pl (P.N.); 2 First Department of Cardiology, Faculty of Medical Sciences in Katowice, Medical University of Silesia, Ziolowa Street 47, 40-635 Katowice, Poland; kmizia-stec@sum.edu.pl (K.M.-S.); afilipecki@sum.edu.pl (A.F.); 3 1st Department of Cardiac Surgery/2nd Department of Cardiology, American Heart of Poland, S. A. Armii Krajowej Street 101, 43-316 Bielsko-Biala, Poland; jolakra@poczta.fm

**Keywords:** *ADAMTS13*, coronary artery disease, long-term survival, polymorphisms

## Abstract

Background: ADAMTS13 is a protein that cleaves large multimers of von Willebrand factor, thereby limiting platelet aggregation and adhesion and regulating thrombogenesis. Research findings suggest a possible association between low ADAMTS13 levels and an increased risk of cardiovascular events, and its activity may be influenced by polymorphic variants of the *ADAMTS13* gene. Methods: The study group included 259 patients diagnosed with coronary artery disease (CAD) and 238 control blood donors. Genotyping of *ADAMTS13* polymorphisms (rs2301612, rs2073932, and rs2285489) was performed using TaqMan PCR. Results: *ADAMTS13* gene polymorphisms showed no association with CAD risk or patient survival at 5- or 10-year follow-up. However, higher HDL cholesterol levels were observed in carriers of the G alleles (rs2301612 and rs2073932) and the T allele (rs2285489). Additionally, the rs2285489 and rs2301612 polymorphisms were associated with certain proatherogenic lipid indices. In silico analysis indicated that all studied polymorphisms influenced gene expression in certain vascular tissues or blood. Conclusions: *ADAMTS13* gene polymorphisms may affect gene expression in specific tissues; however, this effect does not appear sufficient to meaningfully influence CAD onset or patient survival. A significant association between the analyzed polymorphisms and HDL levels or some proatherogenic lipid indices was observed; however, the underlying mechanism requires further investigation.

## 1. Introduction

Coronary artery disease (CAD) is characterized by a diverse pathogenesis and is among the leading global causes of mortality [1]. Therefore, the search for new predictive markers of the disease and patient survival is essential for more effective diagnosis and treatment of individuals with CAD.

ADAMTS13 (A Disintegrin-like And Metalloprotease with Thrombospondin Type 1 Motif, 13) is commonly known as von Willebrand factor-cleaving protein. As a multimeric glycoprotein, von Willebrand factor (VWF) mediates platelet adhesion to the endothelium at sites of vascular wall damage and protects circulating coagulation factor VIII from proteolytic degradation by activated protein C. The primary site of VWF synthesis is the vascular endothelium, from which it is released into the plasma as extremely large multimers with excessive reactivity, impairing their proper function. The regulation of VWF size depends on the metalloproteinase ADAMTS13, which cleaves multimers, reducing platelet adhesion and aggregation, regulating thrombus formation, and inhibiting inflammation [2,3]. The accumulation of extremely large VWF multimers resulting from severe ADAMTS13 deficiency can lead to thrombotic thrombocytopenic purpura (TTP). TTP is a thrombotic microangiopathy in which the formation of platelet-rich microvascular thrombi results in ischemic organ damage, together with severe thrombocytopenia and microangiopathic hemolytic anemia [4].

Previous research indicates that individuals with CAD have higher VWF levels and lower ADAMTS13 antigen values, which are also independent predictors of major adverse cardiovascular and cerebrovascular events [5]. Other studies have demonstrated substantially lower ADAMTS13 levels in individuals with acute myocardial infarction (AMI), with an early decrease in these levels serving as a significant predictor of thrombotic events during one year of follow-up [6].

Polymorphic variants of the *ADAMTS13* gene (9q34.2) may affect protein levels and activity [2,7]. Schettert et al. [8] showed a significant association between the A900V polymorphic variant (rs685523) of the *ADAMTS13* gene and an increased risk of death, especially from cardiac causes. Moreover, patients with chronic coronary syndrome who were heterozygous for another polymorphism, 1342CG (rs2301612), had more clinical endpoints compared to homozygotes (CC and GG) [8]. Some polymorphisms in the introns of the *ADAMTS13* gene (rs2073932, rs652600, rs2285489, rs28793911) were associated with multivessel coronary artery disease, a high Gensini score (reflecting the severity of atherosclerosis), and ischemic stroke in children [2,9]. There are indications that polymorphic variants of the *ADAMTS13* gene, by affecting the levels or activity of this metalloproteinase, could modulate von Willebrand factor activity and thereby influence prothrombotic and proinflammatory tendencies, ultimately impacting the CAD risk and patients’ mortality.

The aim of the study was to analyze the effect of three polymorphic variants of the *ADAMTS13* gene (rs2301612, rs2073932, and rs2285489) on the risk of coronary artery disease, patient survival at 5- and 10-year follow-up, and the risk factor profile.

## 2. Materials and Methods

### 2.1. Study Design

The present study was conducted using a multifaceted approach. It included: (1) a retrospective case–control analysis examining associations between *ADAMTS13* gene polymorphisms and coronary artery disease, including its atherosclerotic phenotype; (2) a prospective evaluation of cardiovascular mortality risk according to *ADAMTS13* gene variants, with 5- and 10-year follow-up; (3) an analysis of the interaction between *ADAMTS13* gene polymorphisms and established coronary risk factors; and (4) an in silico assessment of the potential effect of *ADAMTS13* gene polymorphisms on gene expression. The study was carried out in accordance with STROBE guidelines.

### 2.2. Characteristics of the Study Group

The study included 259 patients with angiographically confirmed premature coronary artery disease (79 women, 180 men), with median age (±QD) 45.50 ± 5.00 years and 238 blood donors as a control group (67 women, 171 men), with median age 44.00 ± 4.00 years, without symptoms of coronary artery disease, history of myocardial infarction (MI), and family history of cardiovascular disease. The group of patients had angiographically confirmed CAD with more than 50% diameter stenosis of at least one major coronary vessel. The coronary angiography was performed by Judkin’s method. The exclusion criteria were: coagulopathy, clinical diagnosis of cardiomyopathy, collagenoses, and acute poisoning (e.g., CO, amphetamine). The study was conducted on biological material collected between 2001 and 2013 and stored at the Department of Biochemistry and Medical Genetics. The study was performed in accordance with the Declaration of Helsinki. Written informed consent was obtained from each study participant, and the study protocol was approved by the Bioethics Committee of the Medical University of Silesia in Katowice (KNW/0022/KB1/17/I/11, NN-013-107/I/00). All participants provided written informed consent before inclusion in the study.

### 2.3. Biochemical Analysis

Serum lipid levels, including triglycerides (TGs), total cholesterol (TC), and high-density lipoprotein (HDL) cholesterol, were measured using colorimetric enzymatic methods (Analco, Warsaw, Poland). The Friedewald formula was used to calculate the serum LDL cholesterol concentration [10].

Proatherogenic lipid indices, including the lipid combination index (LCI) [11], Castelli’s risk index I (CRI-I) [12], Castelli’s risk index II (CRI-II) [12], atherogenic coefficient (AC) [13], atherogenic index of plasma (AIP) [14], and triglyceride-to-HDL-C ratio (TG/HDL-C) [15] were calculated using the following formulas:

LCI [mmol/L] = (TC × TG × LDL-C)/HDL-C

CRI-I [mg/dL] = TC/HDL-C

CRI-II [mg/dL] = LDL-C/HDL-C

AC [mmol/L] = (TC − HDL-C)/HDL-C

AIP [mg/dL] = log_10_ (TG/HDL-C)

TG/HDL [mg/dL] = TG/HDL-C

### 2.4. Genetic Analysis

DNA was isolated from peripheral blood leukocytes using the MasterPure™ DNA purification kit (Epicenter Technologies, Madison, WI, USA). Polymorphic variants of the *ADAMTS13* gene were analyzed using TaqMan^®^ SNP Genotyping Assays (Thermo Fisher Scientific, Waltham, MA, USA). Amplification and genotype reading were performed using the LightCycler 480 Real-Time PCR System (F. Hoffmann-La Roche AG, Basel, Switzerland). The following *ADAMTS13* gene polymorphisms were analyzed: rs2301612, rs2073932, and rs2285489. The first is a missense polymorphism, whereas the other two are located in intronic regions. The polymorphisms were selected based on previously described associations with ADAMTS13 levels (rs2285489) or clinical features (rs2301612, rs2073932), as well as based on the frequency of the minor allele (MAF ≥ 0.20) in populations of European origin.

### 2.5. Statistical Analysis

The obtained data were analyzed using STATISTICA 13.0 software (TIBCO Software Inc., Palo Alto, CA, USA). Quantitative data were assessed for compliance with normal distribution using the Shapiro–Wilk test. For normally distributed data, the means were compared using the *t*-test for independent samples, while in the absence of normal distribution, nonparametric tests were used: the Kruskal–Wallis test, along with *post hoc* analysis (additive model comparing medians of quantitative variables between genotypes); and the Mann–Whitney U test (recessive/dominant model, in which comparisons of medians were made between carriers of one allele and homozygotes for the other allele). Means were presented with their standard deviation (SD), and medians with their quartile deviation (QD) as their spread. The Bonferroni–Hochberg correction was used for multiple testing [16], in which all *p*-values obtained for testing a given research hypothesis were taken into account, for example, whether a specific polymorphism (across all models) was associated with cardio-vascular disease risk factors analyzed in the study (lipid parameters, overweight or obesity, hypertension, diabetes). Allele frequencies were determined based on genotype frequencies. In all groups, the distribution was tested for compliance with the Hardy–Weinberg equilibrium using the *χ*^2^ test. The distribution of all qualitative variables was also compared using the *χ*^2^ test. Yates’ correction was applied to subgroups with fewer than ten subjects. Odds ratios (ORs) and their 95% confidence intervals (CIs) were computed using a univariate analysis.

Statistical significance was set at *p* < 0.050. Cases with missing data were excluded from the relevant comparisons.

### 2.6. Survival Analysis

The endpoint in the current study was death from cardiovascular causes (according to the ICD-10 classification). Data on the causes and dates of death were obtained from the Katowice City Hall and the Central Statistical Office of Poland. The obtained data were analyzed using STATISTICA 13.0 software. Survival curves were analyzed using the Kaplan–Meier estimator, and differences between groups were assessed with the log-rank test. Cox proportional hazards model was used to evaluate the association between genetic variants and survival, with results presented as hazard ratios (HRs) and 95% confidence intervals (CI). The proportional hazards assumptions were evaluated. Statistical significance was set at *p* < 0.05.

### 2.7. In Silico Analysis

For each analyzed single nucleotide polymorphism (SNP), in silico analysis of expression quantitative trait loci (eQTL) was performed using the data obtained from the GTEx (The Genotype-Tissue Expression) Portal [17], to determine whether studied polymorphisms affect *ADAMTS13* expression. Expression in the aorta, coronary arteries, tibial artery, and whole blood was analyzed.

## 3. Results

### 3.1. General Characteristics of the Study Groups

Patients with CAD were characterized by a significantly higher prevalence of hypertension, diabetes mellitus, cigarette smoking, and overweight/obesity (BMI ≥ 25), while the prevalence of obesity (BMI ≥ 30) did not differ between the groups (Table 1). Despite receiving lipid-lowering pharmacotherapy, patients with CAD also had higher levels of all lipid profile parameters—except for HDL levels, which were significantly lower in the CAD group—as well as higher values of lipid atherogenic indices compared to the controls (Table 1).

### 3.2. Analysis of the Association Between ADAMTS13 Gene Polymorphisms and Coronary Artery Disease

The distribution of genotypes and alleles for all studied polymorphisms was consistent with the Hardy–Weinberg equilibrium (*p* > 0.05). Statistical analysis did not reveal any significant associations between the studied polymorphisms and coronary artery disease (Table 2). Despite the lack of statistical significance, slightly higher frequencies of certain alleles were observed in the CAD patient group compared to the control group for all polymorphisms (rs2301612: allele C, 56.90% vs. 54.90%; rs2073932: allele A, 42.90% vs. 40.10%; rs2285489: allele C, 61.80% vs. 58.20%). In addition, no significant associations were observed between the studied polymorphisms and myocardial infarction or degree of coronary artery stenosis and severity of atherosclerosis (multi-vessel disease). The results presented were obtained from univariate analysis. A multivariate analysis was not performed, as only variables showing a *p*-value below 0.250 in univariate analysis were considered eligible for inclusion in the model [18,19]. Since no statistically significant differences in the distribution of the analyzed polymorphic variants between groups were observed, further multivariate modeling was not undertaken.

### 3.3. Analysis of the Association Between ADAMTS13 Gene Polymorphisms and Patients’ Survival

**Figure 1:**
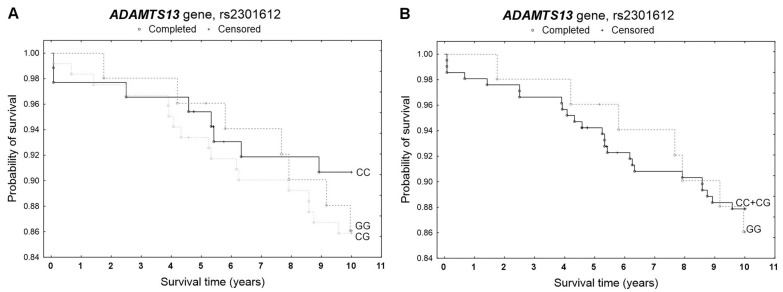
Kaplan–Meier survival curves for CAD patients by rs2301612 (**A**) additive model: for 5-year survival, *χ*^2^ = 0.669, *p* = 0.716; for 10-year survival, *χ*^2^ = 1.010, *p* = 0.603. (**B**) recessive model: for 5-year survival, the log-rank statistic was 0.528, *p* = 0.597; for 10-year survival, the log-rank statistic was −0.278, *p* = 0.781.

**Figure 2:**
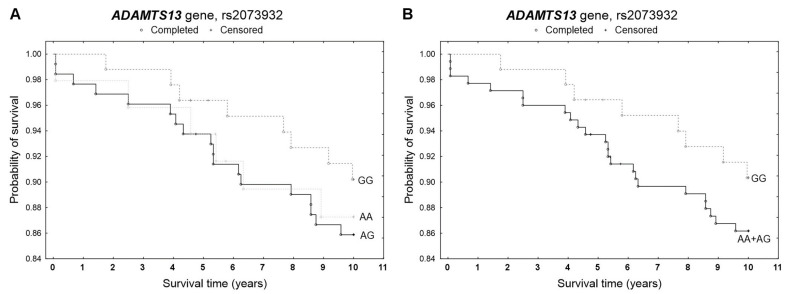
Kaplan–Meier survival curves for CAD patients by rs2073932 (**A**) additive model: for 5-year survival, *χ*^2^ = 0.807, *p* = 0.668; for 10-year survival *χ*^2^ = 1.016, *p* = 0.602. (**B**) dominant model: for 5-year survival, the log-rank statistic was 0.913, *p* = 0.361; for 10-year survival, the log-rank statistic was 0.980, *p* = 0.327.

**Figure 3:**
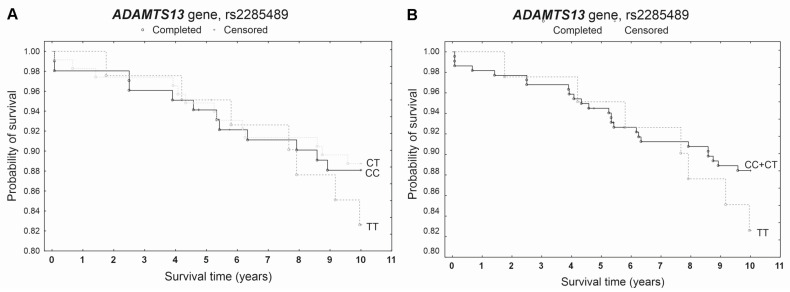
Kaplan–Meier survival curves for CAD patients by rs2285489 (**A**) additive model: for 5-year survival, *χ*^2^ = 0.089, *p* = 0.956; for 10-year survival, *χ*^2^ = 0.781, *p* = 0.677. (**B**) dominant model: for 5-year survival, the log-rank statistic was 0.169, *p* = 0.865; for 10-year survival, the log-rank statistic was −0.948, *p* = 0.343.

During the 5-year follow-up, 14 deaths were recorded among 259 patients, while a total of 32 deaths occurred during the 10-year follow-up. None of the studied polymorphisms showed a statistically significant effect on patient survival at either 5 or 10 years in the Kaplan–Meier analysis (Figure 1, Figure 2 and Figure 3). A slightly increased survival probability throughout the entire analyzed period was observed only in GG homozygotes of the rs2073932 polymorphism compared with A allele carriers (Figure 2). In the multivariable Cox proportional hazards model adjusted for age and sex, genotype was also not significantly associated with survival. The proportional hazards assumption was violated only for rs2301612 in the additive model during the 10-year follow-up (Table 3).

### 3.4. Analysis of the Associations of ADAMTS13 Gene Polymorphisms and CAD Risk Factors

**Figure 4:**
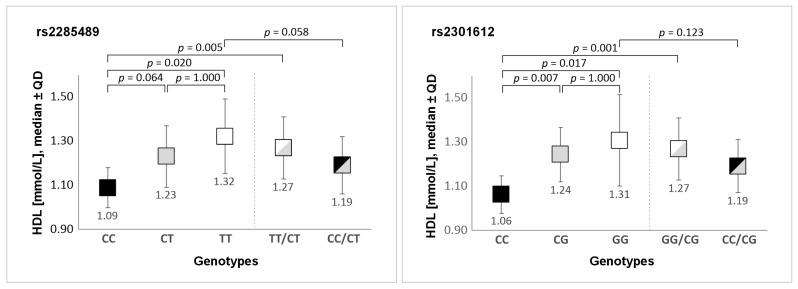
Comparison of genotype variants of rs2285489 and rs2301612 polymorphisms with HDL cholesterol levels. Legend: QD, Quartile Deviation.

Analyses were performed to examine the association between *ADAMTS13* gene polymorphism variants and traditional CAD risk factors, including hypertension, overweight/obesity (BMI ≥ 25), diabetes, and lipid metabolism parameters (triglycerides, LDL cholesterol, HDL cholesterol, total cholesterol). Due to the fact that many patients with CAD were treated with drugs that affect lipid levels or blood pressure, the analyses were performed only for the control group. The only factor that showed a significant association with *ADAMTS13* gene polymorphisms was HDL cholesterol level. Elevated HDL cholesterol levels were observed in TT homozygotes and T allele carriers (TT/CT genotypes) of the rs2285489, compared to CC homozygotes (*p* = 0.020 and *p =* 0.005, respectively). Similarly, subjects with GG and GG/CG genotypes (rs2301612) had elevated HDL cholesterol levels compared to the CC homozygotes (*p* = 0.017 and *p* = 0.001, respectively). The results are presented in Figure 4. Additionally, carriers of the G allele of the rs2073932 polymorphism had also significantly higher HDL cholesterol levels than AA homozygotes (1.22 ± 0.25 vs. 1.04 ± 0.19, respectively, *p* = 0.034). After adjustment for multiple comparisons, the differences remained statistically significant for rs2285489 (T allele carriers vs. CC homozygotes) and for rs2301612 (G allele carriers vs. CC homozygotes).

In addition to comparing lipid profiles across genotype variants, we assessed *ADAMTS13* gene polymorphisms in relation to proatherogenic lipid indices, including LCI, CRI-I, CRI-II, AC, AIP, and TG/HDL. Notably, patients with CAD, despite receiving lipid-lowering therapy, exhibited significantly higher values of all lipid indices analyzed in this study compared with the control group (Table S1). For the rs2285489 polymorphism, CC homozygotes exhibited significantly higher (*p* < 0.050) values of proatherogenic lipid indices—including CRI-I, AC, AIP, and TG/HDL-C—compared with T allele carriers (TT/CT). Likewise, CC homozygotes of rs2301612 showed elevated values across all analyzed indices relative to G allele carriers (GG/CG). For both polymorphisms, the strongest and most statistically significant differences were observed for AIP and TG/HDL (Table 4). Under the additive model, no statistically significant differences were found between genotypes of any of the analyzed polymorphisms. The differences that remained statistically significant after adjustment for multiple comparisons are marked in Table 4.

### 3.5. In Silico Analysis of the Impact of ADAMTS13 Gene Polymorphisms on Gene Expression

**Figure 5:**
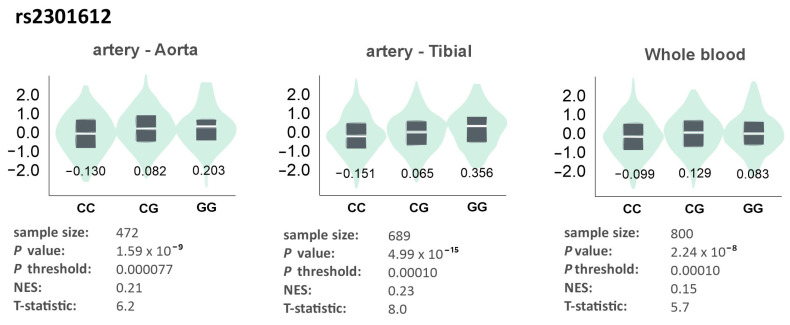
Gene expression of *ADAMTS13* in different tissues dependently on the genotype of the rs2301612 polymorphism. The values on the charts represent the medians for the individual genotypes. Based on the GTEx Portal [17].

An in silico analysis using the GTEx Portal tool [17] demonstrated the influence of all studied polymorphisms on gene expression in specific tissues. The rs2301612 polymorphism affected expression in the aorta, tibial artery, and whole blood, with GG homozygotes showing the highest expression in the aorta and tibial artery, and CG heterozygotes in whole blood (Figure 5).

**Figure 6:**
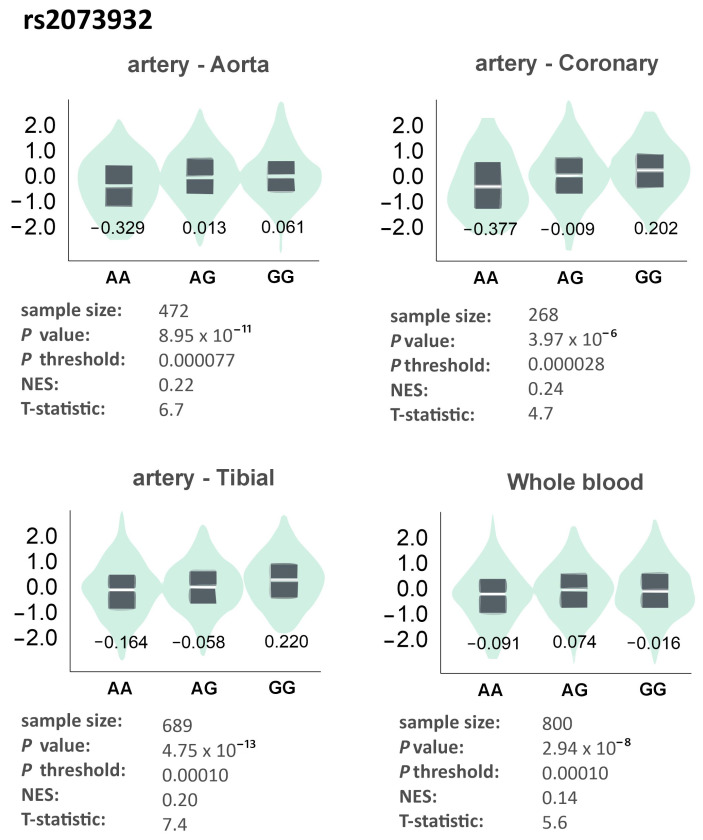
Gene expression of *ADAMTS13* in different tissues dependently on the genotype of the rs2073932 polymorphism. The values on the charts represent the medians for the individual genotypes. Based on the GTEx Portal [17].

In the four tissues listed above, gene expression was also dependent on the rs2073932 polymorphism. In each of them, except for whole blood, expression was highest in GG homozygotes (Figure 6).

**Figure 7:**
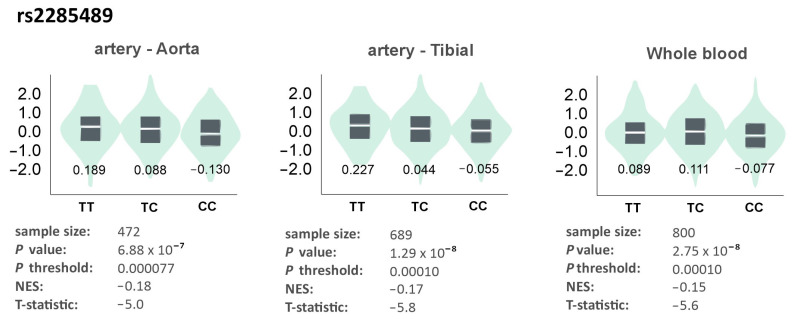
Gene expression of *ADAMTS13* in different tissues dependently on the genotype of the rs2285489 polymorphism. The values on the charts represent the medians for the individual genotypes. Based on the GTEx Portal [17].

The rs2285489 polymorphism was associated with gene expression in the aorta and tibial artery (highest expression in TT homozygotes) and in whole blood (highest expression in CT heterozygotes) (Figure 7).

## 4. Discussion

Our research identified associations between *ADAMTS13* polymorphisms and HDL cholesterol levels or some proatherogenic lipid indices in the control group not undergoing lipid-lowering therapy. Carriers of the TT and TT/CT (rs2285489) genotypes, GG and GG/CG (rs2301612) genotypes, as well as the GG/GC genotype of the rs2073932 polymorphism, had significantly higher HDL cholesterol levels. After adjustment for multiple comparisons, the differences remained statistically significant for the rs2285489 SNP (T allele carriers vs. CC homozygotes) and for rs2301612 (G allele carriers vs. CC homozygotes). Additionally, in silico analysis suggested that all analyzed polymorphisms influence gene expression in certain tissues. The highest expression in the aorta and tibial artery was observed for rs2285489 (TT), rs2301612 (GG), and rs2073932 (GG) genotypes, and individuals with the GG genotype (rs2073932) also showed the highest expression in coronary vessels. The impact of specific genetic variants on gene expression could be one explanation for the observed associations between *ADAMTS13* polymorphisms and HDL cholesterol levels. However, the results of the in silico analysis using the GTEx database should be interpreted with caution, as they are solely correlative in nature and require experimental validation. Such analysis may support associations between specific genotypes and gene expression (eQTL effects) in selected tissues; however, it does not directly demonstrate changes in ADAMTS13 protein levels in the studied cohort.

The observed associations between *ADAMTS13* polymorphisms and HDL levels may be partly related to broader biological processes involving vascular inflammation and lipid metabolism; however, these mechanisms remain speculative in the context of our study. To date, the influence of the polymorphisms analyzed in this study on HDL levels has not been investigated. However, Schooling et al. [20] found no association between genetically predicted ADAMTS13 activity and HDL levels. In contrast, the few studies that have examined the relationship between ADAMTS13 activity and HDL levels are inconsistent with our observations. In a case–control study involving individuals who had suffered a myocardial infarction, a negative correlation was observed between ADAMTS13 and HDL cholesterol levels in the control group [21]. Similar results were reported in a study of patients with aortic valve stenosis [22] and in hemodialysis patients [23]. These discrepancies may result from differences in study methodology, analyzed populations, disease prevalence, patient clinical profile, pharmacotherapy, or other factors that may modulate ADAMTS13 activity and lipid profiles. It is worth noting that our analysis focused solely on blood donors, as most patients were treated with lipid-lowering medications, which could have influenced the results. Therefore, this approach limits the generalizability of the findings to the CAD group and reduces direct comparability with studies conducted in patient populations.

Our research also revealed significant associations between the *ADAMTS13* rs2285489 and rs2301612 polymorphisms and several proatherogenic lipid indices. These indices are better predictors of atherosclerotic risk than individual lipid parameters, as they reflect both the quality and balance of the lipid profile. After adjustment for multiple testing, statistically significant associations were confirmed for AIP and TG/HDL. Although the available literature lacks reports on associations between *ADAMTS13* polymorphisms and these lipid indices, the observed trend is consistent with other findings of the present study. Nevertheless, many studies indicate that indices such as AIP, TG/HDL, CRI-I, CRI-II, AC, and LCI are associated with the risk of cardiovascular disease, including CAD [11,13,24,25,26,27], which has also been confirmed in our study. However, this result, together with the higher prevalence of dyslipidemia in the CAD group, should be interpreted with caution, since many subjects with CAD were treated with lipid-lowering drugs.

In our study, no significant association was found between the analyzed polymorphisms and coronary artery disease, myocardial infarction, degree of coronary artery stenosis, severity of atherosclerosis, or patient survival at 5- and 10-year follow-up. This lack of association was somewhat unexpected, as our in silico analysis suggests an effect of all analyzed polymorphisms on gene expression, and some of the analyzed polymorphisms (e.g., rs2285489) were previously reported to affect ADAMTS13 levels [2]. The protein level, in turn, was significantly associated with acute myocardial infarction, and an early decrease in ADAMTS13 levels was also a significant predictor of future thrombotic events [6]. Another polymorphism studied, namely rs2301612, was associated with the number of clinical endpoints in patients with chronic coronary syndrome [8]. The rs2073932 and rs2285489 polymorphisms were also associated with multivessel coronary artery disease and a high Gensini score [2,9]. Notably, rs2301612 and rs2285489 have additionally been linked to an increased risk of cerebral aneurysm, indicating their potential role in vascular pathology beyond CAD [28]. Furthermore, the haplotype H4, including the rs2073932, was associated with a lower risk of CAD in a Thai cohort, suggesting a potential protective role of this haplotype against the disease [29].

It therefore appears that all the polymorphisms we analyzed in this study may influence gene expression in specific tissues; however, this effect is not strong enough to demonstrate a statistically significant association with clinical features or survival, and may also depend on the genetic and environmental context.

The main limitation of our study was the relatively modest sample size, which, together with the low number of fatal events, may have reduced statistical power, particularly in survival analysis. However, the sample size was determined by our aim to include an ethnically homogeneous cohort of individuals with premature coronary artery disease, in whom genetic factors may play a larger role. Moreover, we did not assess ADAMTS13 levels or activity, which could have provided more definitive conclusions; instead, we performed in silico analysis of the genotype-dependent effects of *ADAMTS13* polymorphisms on gene expression.

## 5. Conclusions

In conclusion, the studied polymorphisms may affect gene expression in specific tissues; however, this effect does not appear sufficient to meaningfully influence CAD patient survival or disease onset. A clear association between the analyzed polymorphisms and HDL levels, as well as proatherogenic lipid indices, was observed; however, the underlying mechanism of this association requires further investigation.

## Figures and Tables

**Table 1:** Risk factors of CAD in patients and controls (blood donors).

Characteristics	CAD Patients			Controls			*p*
	n*	*n*	%	n*	*n*	%	
N		259			238		
Male sex	259	180	69.50	238	171	71.85	0.565
Hypertension	255	205	80.39	105	9	8.57	0.000
DM	259	7	2.70	238	0	0.00	0.030
Cigarette smoking	219	143	65.30	231	66	28.45	0.000
Overweight/obesity (BMI ≥ 25)	224	162	72.32	180	110	61.11	0.017
Obesity (BMI ≥ 30)	224	50	22.32	180	34	18.89	0.398
	n*	Median	±QD	n*	Median	±QD	
Age [years]	251	45.50	5.00	228	44.00	4.00	0.071
BMI [kg/m^2^]	238	26.70	2.60	200	25.40	2.60	0.016
TC [mmol/L]	246	5.80	0.92	200	5.21	0.74	0.000
HDL [mmol/L]	242	1.06	0.16	199	1.22	0.25	0.000
LDL [mmol/L]	243	4.25	0.87	199	3.62	0.82	0.000
TG [mmol/L]	245	1.81	0.53	199	1.40	0.47	0.000
LCI [mmol/L]	242	38.25	27.12	197	22.56	14.94	0.000
CRI-I [mg/dL]	242	5.27	1.10	197	4.40	1.02	0.000
CRI-II [mg/dL]	242	3.46	0.88	197	2.71	0.90	0.000
AC [mmol/L]	242	4.27	1.10	197	3.40	1.02	0.000
AIP [mg/dL]	242	0.59	0.17	197	0.44	0.20	0.000
TG/HDL [mg/dL]	242	3.90	1.43	197	2.75	1.26	0.000

Legend: AC, atherogenic coefficient; AIP, atherogenic index of plasma; BMI, body mass index; CRI, Castelli’s risk index; DM, diabetes mellitus; HDL, high-density lipoprotein cholesterol; LCI, lipid combination index; LDL, low-density lipoprotein cholesterol; TC, total cholesterol; TG, triglycerides; n*—number of subjects in respective analyses.

**Table 2:** Genotype and allele frequencies of the *ADAMTS13* gene in CAD patients and controls.

Genotype/ Allele	CAD Patients *n* (%)	Controls *n* (%)	Inheritance Model	OR (95% CI)	*p*
rs2301612					
CC	87 (33.60)	70 (29.50)	Dominant, vs. CG + GG	1.21 (0.83–1.76)	0.332
CG	121 (46.70)	120 (50.60)	Additive, vs. CC	0.81 (0.54–1.21)	0.309
GG	51 (19.70)	47 (19.80)	Recessive, vs. CC + CG	0.99 (0.64–1.54))	0.969
C	295 (56.90)	260 (54.90)	-	1.09 (0.85–1.40)	0.506
G	223 (43.10)	214 (45.10)	-	0.92 (0.71–1.18)	0.506
rs2073932					
GG	84 (32.40)	86 (36.10)	Dominant, vs. AA + AG	1.85 (0.59–1.23)	0.385
AG	128 (49.40)	113 (47.50)	Additive, vs. GG	1.16 (0.78–1.72)	0.460
AA	47 (18.20)	39 (16.40)	Recessive, vs. GG + AG	0.88 (0.55–1.40)	0.604
G	296 (57.10)	285 (59.90)	-	0.89 (0.68–1.15)	0.383
A	222 (42.90)	191 (40.10)	-	1.12 (0.87–1.44)	0.383
rs2285489					
CC	102 (39.40)	84 (35.30)	Dominant, vs. CT + TT	0.84 (0.58–1.21)	0.347
CT	116 (44.80)	109 (45.80)	Additive, vs. CC	0.88 (0.59–1.29)	0.507
TT	41 (15.80)	45 (18.90)	Recessive, vs. CC + CT	0.81 (0.51–1.28)	0.365
C	320 (61.80)	277 (58.20)	-	1.16 (0.90–1.50)	0.249
T	198 (38.20)	199 (41.80)	-	0.86 (0.67–1.11)	0.249

Legend: OR, odds ratio; CAD, coronary artery disease (patient group).

**Table 3:** Results of the Cox proportional hazards model with verification of the proportional hazards assumption.

Inheritance Model	Follow-Up	*p*	HR (95%CI)	*p* for PH
rs2301612				
Additive	5 years 10 years	0.729 0.516	1.15 (0.53–2.48) 0.85 (0.51–1.40)	0.292 0.049 *
Recessive	5 years 10 years	0.567 0.734	1.56 (0.34–7.08) 0.86 (0.37–2.03)	0.646 0.099
rs2073932				
Additive	5 years 10 years	0.344 0.477	1.45 (0.67–3.14) 1.20 (0.72–2.00)	0.458 0.088
Dominant	5 years 10 years	0.342 0.393	1.88 (0.51–6.95) 1.43 (0.63–3.23)	0.333 0.179
rs2285489				
Additive	5 years 10 years	0.592 0.637	1.24 (0.56–2.77) 0.89 (0.54–1.47)	0.469 0.090
Dominant	5 years 10 years	0.894 0.290	1.11 (0.24–5.02) 0.63 (0.27–1.48)	0.665 0.122

Legend: *, differences statistically significant; HR, hazard ratio; PH, proportional hazards.

**Table 4:** Values of proatherogenic lipid indices according to *ADAMTS13* gene polymorphic variants.

**rs2285489**	**Parameter**	**CC, *n* = 69**		**TT/CT, *n* = 128**		***p***
		median	±QD	median	±QD	
	LCI [mmol/L]	27.04	18.30	21.15	14.65	0.075
	CRI_I [mg/dL]	4.53	0.95	4.29	1.09	0.045
	CRI_II [mg/dL]	2.90	0.84	2.62	0.97	0.096
	AC [mmol/L]	3.53	0.95	3.29	1.09	0.045
	AIP [mg/dL]	0.52	0.13	0.39	0.21	0.007 *
	TG/HDL [mg/dL]	3.33	0.98	2.43	1.19	0.007 *
**rs2301612**	**Parameter**	**CC, *n* = 58**		**GG/CG, *n* = 138**		***p***
		median	±QD	median	±QD	
	LCI [mmol/L]	26.15	17.38	21.15	14.66	0.040
	CRI_I [mg/dL]	4.55	0.97	4.29	1.06	0.013
	CRI_II [mg/dL]	2.85	0.81	2.62	0.97	0.033
	AC [mmol/L]	3.55	0.97	3.29	1.06	0.013
	AIP [mg/dL]	0.53	0.13	0.40	0.21	0.003 *
	TG/HDL [mg/dL]	3.37	0.98	2.51	1.13	0.003 *

Legend: AC, atherogenic coefficient; AIP, atherogenic index of plasma; CRI, Castelli’s risk index; LCI, lipid combination index; HDL, high-density lipoprotein cholesterol; TG, triglycerides; QD, quartile deviation; *, differences remaining significant after Bonferroni–Hochberg correction for multiple comparisons.

## Data Availability

The data supporting the findings of this study are available within the article and its Supplementary Materials. Individual-level data are not publicly available due to ethical and privacy restrictions but may be obtained from the corresponding author upon reasonable request and subject to approval by the relevant ethics committee.

## Supplementary Materials

The following supporting information can be downloaded at https://www.mdpi.com/article/10.3390/genes17050508/s1, Table S1: Proatherogenic lipid indices in CAD patients and in the blood donors group.

## Abbreviations

The following abbreviations are used in this manuscript:

CAD	Coronary artery disease
VWF	Von Willebrand factor
AMI	Acute myocardial infarction
MI	Myocardial infarction
TTP	Thrombotic thrombocytopenic purpura
TG	Triglycerides
TC	Total cholesterol
HDL	High-density lipoprotein
LCI	Lipid combination index
CRI-I	Castelli’s risk index I
CRI-II	Castelli’s risk index II
AC	Atherogenic coefficient
AIP	Atherogenic index of plasma
TG/HDL-C	Triglyceride-to-HDL-C ratio
SD	Standard deviation
QD	Quartile deviation
OR	Odds ratios
CI	Confidence intervals
SNP	Single nucleotide polymorphism
eQTL	Expression quantitative trait loci
ApoA1	Apolipoprotein A-I
