# Supplementary material for: ADAMTS13 Gene Polymorphisms and Coronary Artery Disease Risk, Long-Term Survival, and Risk Factor Profile
Source: Genes (Basel). 2026 Apr 25;17(5):508. doi: 10.3390/genes17050508 (PMC13205630; doi:10.3390/genes17050508)
Supplement: Supplementary file 1 [file genes-17-00508-s001.zip › Table S1.pdf]

Table S1. Pro-atherogenic lipid indices in CAD patients and in the blood donors group.

Parameter	CAD, n=257		Blood donors, n=238		*p*
	**median**	**± QD**	**median**	**± QD**	
LCI [mmol/L]	37.80	27.13	25.19	17.94	<0.001
CRI_I [mg/dL]	5.23	1.07	4.60	1.09	<0.001
CRI_II [mg/dL]	3.43	0.88	2.92	0.92	<0.001
AC [mmol/L]	4.23	1.07	3.60	1.09	<0.001
AIP [mg/dL]	0.58	0.17	0.47	0.20	<0.001
TG/HDL [mg/dL]	3.79	1.39	2.98	1.33	<0.001

Legend: QD – Quartile Deviation, CAD—coronary artery disease (patient group).
